# Integrated gut microbiota and metabolome analyses link anger to metabolic dysregulation in patients with type 2 diabetes mellitus

**DOI:** 10.3389/fmicb.2026.1797695

**Published:** 2026-07-09

**Authors:** Xue-li Bao, Ye-xin Chen, Tu-nan Ding, Dan-dan Zhao, Mo-han Sun, Qian-wen Yang, Dong-sen Hu, Fang-fang Mo, Gui-xiao Yang, Si-hua Gao, Jin-kun Ma, Tian Tian

**Affiliations:** 1 Dongzhimen Hospital, Beijing University of Chinese Medicine, Beijing, China; 2 Beijing University of Chinese Medicine, Beijing, China; 3 China Press of Chinese Medicine, Beijing, China; 4 China Academy of Chinese Medical Sciences, Beijing, China

**Keywords:** anger, gut microbiota, metabolic dysregulation, metabolomics, type 2 diabetes

## Abstract

**Objective:**

This exploratory pilot study aimed to investigate the associations of anger-related irritability symptoms with gut microbiota and circulating metabolites in patients with type 2 diabetes mellitus (T2DM) using multi-omics analysis.

**Methods:**

We conducted a cross-sectional study, in which T2DM patients were categorized into a self-reported irritable T2DM group (IDM, *n* = 29) and a self-reported non-irritable DM group (NIDM, *n* = 28) based on Visual Analog Scale (VAS) scores, with a healthy control group (HC, *n* = 30) also established. Fecal 16S rRNA gene sequencing and UPLC-MS/MS-based untargeted metabolomic profiling of blood samples were performed. Differences in microbial community structure between groups were analyzed using alpha and beta diversity metrics and linear discriminant analysis effect size (LEfSe) analysis. Differentially abundant genera were identified by MaAsLin2 with adjustment for confounders. Exploratory metabolite markers were screened based on thresholds for *p* < 0.05, variable importance in projection (VIP) > 1, |log_2_ fold change (FC)| > 1. Associations between differentially abundant genera and exploratory metabolite markers were explored using Spearman correlation analysis. Functional prediction was conducted based on differentially represented KEGG orthology (KO) and clusters of orthologous groups (COG) entries to identify altered metabolic pathways.

**Results:**

Both beta diversity and LEfSe analyses revealed differences in gut microbial community structure and potential discriminatory taxa among the three groups. Five differentially abundant genera and twelve exploratory metabolite markers were identified between IDM and NIDM. A combined model incorporating microbial and metabolomic markers demonstrated superior diagnostic performance (AUC = 0.872) compared with models using either type of marker alone. Twelve statistically significant microbiota–metabolite associations were found in Spearman analysis. Functional prediction analysis indicated enrichment of bile acid and tryptophan metabolism pathways.

**Conclusion:**

VAS-defined irritability symptoms were associated with specific gut microbial and metabolomic alterations in T2DM, providing exploratory evidence for future studies on emotion-related metabolic dysregulation.

## 1 Introduction

The comorbidity of metabolic diseases and psychiatric disorders has become an important focus of modern medical research (Guo et al., 2025; Vancampfort et al., 2015), particularly among patients with type 2 diabetes mellitus (T2DM)(Pan et al., 2025). Large-scale epidemiological studies have shown that the prevalence of depression in T2DM patients is significantly higher than in the general population. A meta-analysis involving over 17,000 patients revealed a comorbidity rate of approximately 35% (Yang et al., 2025), with this dual diagnosis further impairing glycemic control, increasing cardiovascular mortality, and elevating the overall risk of death (Yang et al., 2025; Zhu et al., 2022). Accordingly, growing attention has been paid to the comorbidity of diabetes with depression and anxiety disorders in recent years. Most existing studies have focused on how various mental health disorders affect the onset, progression, and risk of complications in diabetes, highlighting the importance of improving disease management for diabetes patients with mental comorbidities (Fanelli et al., 2025; Al-Jabi, 2024).

However, among the broad spectrum of negative emotions, affective states such as anger and hostility, which are common yet do not necessarily meet the diagnostic criteria for psychiatric disorders, have been relatively neglected with regard to their impact on diabetic patients. Contemporary literature suggests that anger frequently co-occurs with depressive and anxiety symptoms, yet remains a partially distinct construct that should not be simply equated with categorical psychiatric diagnoses (Perlis et al., 2024). As a key component of the stress-response system, the neuroendocrine-immune network is highly sensitive to fluctuations in negative emotions (Xie et al., 2025). Importantly, anger in the present study was not conceptualized as a formal psychiatric disorder. Rather, it was defined as a clinically relevant negative affective dimension that may cut across multiple psychiatric and medical conditions. Research has shown that subclinical anger and hostility, which do not meet diagnostic thresholds for mental disorders, may contribute to metabolic disturbances by activating the hypothalamic–pituitary–adrenal (HPA) axis, enhancing physiological stress responses, and triggering low-grade inflammation (Staicu and Cutov, 2010; Pouwer et al., 2010). Consistently, high levels of hostility in patients with diabetes have been associated with more pronounced physiological responses to acute stress (Hackett et al., 2015). Therefore, our focus on anger was intended to examine a transdiagnostic psychophysiological symptom dimension that may interact with the gut–brain–metabolic axis, rather than to implicitly classify anger as a psychiatric disorder (Vidal-Ribas et al., 2016).

In the general population, an angry disposition is linked to poor glucose regulation (Tsenkova et al., 2014). However, existing literature has not sufficiently explored the biological mechanisms by which anger may influence diabetes (Ward et al., 2024; Mohseni et al., 2023). In particular, little is known about how anger specifically affects the metabolic microenvironment, immune homeostasis, and the gut ecosystem in diabetic patients.

To address this gap, the present exploratory study innovatively focused on T2DM patients with self-reported anger-related irritability and employed a multi-omics strategy integrating 16S rRNA gut microbiome sequencing with untargeted metabolomics analysis. This approach enabled a systematic comparison of multidimensional differences among self-reported irritable diabetic patients, self-reported non-irritable diabetic patients and healthy controls. Through this study, we aimed to characterize the specific microbiome-metabolite co-variation patterns associated with self-reported anger, providing exploratory evidence for potential mechanisms linking emotional fluctuations to metabolic disturbances in diabetes and offering insights into the role of negative emotions in metabolic disease progression.

## 2 Methods

### 2.1 Study design and participants

This cross-sectional study enrolled individuals with a diagnosis of T2DM at Dongzhimen Hospital between March 2023 and February 2024. All participants were rigorously screened. Self-reported anger-related irritability was assessed using the visual analog scale (VAS), a widely used instrument for rapid assessment of subjective mood and pain intensity. Its simple and intuitive design allows for rapid assessment of emotional fluctuations. Prior studies have confirmed that the VAS is an efficient and convenient instrument for assessing emotional states. Although emotional disorders such as depression and anxiety are commonly assessed using multidimensional scales, anger is not defined as a discrete psychiatric disorder, and no universally accepted instrument is available for its specific measurement. In this study, “anger” was operationalized as self-perceived anger/irritability intensity over the preceding 3 months, rather than as a DSM-defined disorder or a stable personality trait. The single-dimensional nature of the VAS enables participants to quickly recall and subjectively assess the frequency of anger, making it suitable for exploratory stratification in this study (Cloos et al., 2025; Dutheil et al., 2024; Moulton et al., 2021). Scores ranged from 0 to 10, where 0 represented “never feeling angry” and 10 indicated “feeling angry very frequently.” Participants were asked to recall whether they had frequently felt angry during the past three months. Individuals scoring 7–10 were classified into the self-reported irritable DM (IDM) group, while those scoring 0–3 were placed in the self-reported non-irritable DM (NIDM) group.

**Figure 1:**
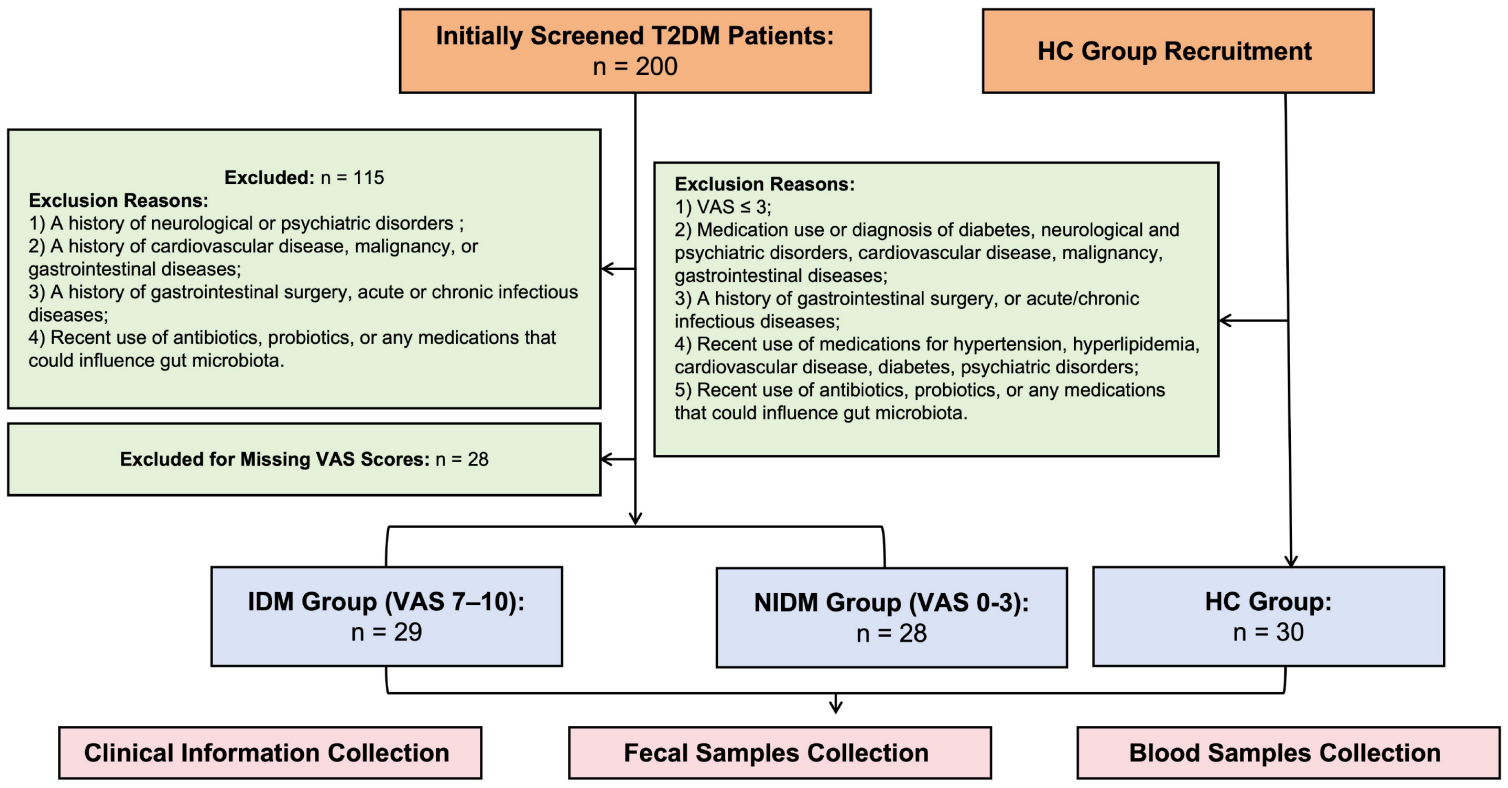
The study flow chart.

Before enrollment, a total of 200 T2DM patients were screened to exclude neurological or psychiatric disorders, including stroke, depression, anxiety, Alzheimer's disease, and Parkinson's disease. Participants with a history of cardiovascular disease, malignancy, or gastrointestinal diseases (e.g., chronic hepatitis, cholecystitis, pancreatitis, irritable bowel syndrome, or inflammatory bowel disease) were also excluded. Additional exclusion criteria included a history of gastrointestinal surgery, acute or chronic infectious diseases, or recent use (within 3 months) of antibiotics, probiotics, or any medications that could influence gut microbiota. Based on these criteria, 115 patients were excluded, and another 28 were excluded because of missing VAS scores for anger. Ultimately, 29 patients with VAS scores of 7–10 were assigned to the IDM group, and 28 patients with VAS scores of 0–3 were assigned to the NIDM group. The HC group (*n* = 30) was recruited separately from routine health checkups during the same period (Figure 1). All HC participants had VAS scores ≤ 3 and had no medication use or diagnosis of diabetes, neurological/psychiatric disorders, cardiovascular disease, malignancy, gastrointestinal diseases, history of gastrointestinal surgery, or acute/chronic infectious diseases. Specifically, none of the HC participants were taking medications for hypertension, hyperlipidemia, cardiovascular disease, diabetes, psychiatric disorders, and none had used antibiotics, probiotics, or any medications that could influence the gut microbiota within the past 3 months. Baseline characteristics are presented as mean (standard deviation) for continuous variables and as frequencies and percentages for categorical variables. Group comparisons for continuous variables were performed using Welch's *t*-test or ANOVA. For between-group comparisons of categorical variables, Fisher's exact test was used when expected frequencies were < 5; otherwise, the chi-squared test was applied. Demographic and clinical data were collected from electronic medical records, including patient history, laboratory findings, and medication use. This study was approved by the Ethics Committee of Dongzhimen Hospital (Approval No. 2022DZMEC-461-02). Written informed consent was obtained from all participants prior to enrollment.

### 2.2 Sample collection and processing

Fecal samples were immediately refrigerated and transported to the laboratory, where they were snap-frozen in liquid nitrogen and stored at −80 °C for subsequent 16S rRNA gene sequencing analysis. Total DNA was extracted using a magnetic bead-based fecal genomic DNA extraction kit (TIANGEN BIOTECH, Beijing, China). The extraction procedure included mechanical lysis, magnetic bead binding, washing steps, and final elution. DNA purity and concentration were assessed by 1% agarose gel electrophoresis, and DNA was subsequently diluted to 1 ng/μL with sterile water for PCR amplification. The V4 region of the bacterial 16S rRNA gene was targeted using specific primers 515F/806R and Phusion^®^ High-Fidelity PCR Master Mix (New England Biolabs, USA). PCR products were verified by 2% agarose gel electrophoresis, purified using the corresponding magnetic bead-based purification kit, quantified, and pooled in equal amounts. Sequencing libraries were constructed using the NEB Next^®^ Ultra™ II FS DNA PCR-free Library Prep Kit (New England Biolabs, USA). The library preparation included end-repair, A-tailing, adapter ligation, and a final clean-up step. The libraries were quantified by Qubit (Thermo Fisher, USA) and real-time PCR to ensure accurate concentration. Finally, sequencing was performed on the Illumina NovaSeq 6000 platform (Illumina, San Diego, CA, USA) with PE250 paired-end sequencing (Degnan and Ochman, 2012; Caporaso et al., 2012). All procedures followed standard laboratory protocols to ensure library quality and analytical reliability.

### 2.3 Gut microbiota analysis

Raw sequencing data were first demultiplexed according to barcode sequences and PCR amplification primers. Paired-end reads were assembled using FLASH (version 1.2.11) to obtain raw tags (Magoč and Salzberg, 2011), followed by strict quality filtering with fastp (version 0.23.1) to remove reads containing >10% ambiguous bases or >50% low-quality bases (Bokulich et al., 2013). Chimeric sequences were then identified and removed using UCHIME against the Silva or Unite database (Edgar et al., 2011). Sequence processing was performed using QIIME2 (Bolyen et al., 2019), and denoising was conducted with the DADA2 plugin to generate amplicon sequence variants (ASVs) (Callahan et al., 2016). Taxonomic annotation of ASVs was performed using QIIME2 against the Silva 138.1 database. To reduce the impact of low-frequency noise, the minimum prevalence threshold for excluding low-abundance taxa was set at 10% (Huang et al., 2026). Data were then rarefied to the minimum sequencing depth across samples to normalize sequencing effort. Using the normalized ASV table, alpha diversity was assessed based on the Chao1, Shannon, and Simpson indices, which were computed using the “*phyloseq*” package in R. Specifically, Chao1 estimates species richness, Shannon reflects both richness and evenness, and Simpson emphasizes dominance. Group differences in alpha diversity were examined via the Kruskal–Wallis test. Beta diversity was analyzed using principal coordinates analysis (PCoA) based on the weighted UniFrac distance matrix, computed using the “*vegan*” package in R. This distance metric takes into account both the phylogenetic relationships and the relative abundances of microbial taxa. Permutational multivariate analysis of variance (PERMANOVA) was performed to calculate *R*^2^ and *p*-values. A higher *R*^2^ indicated that group status explained a greater proportion of variation in microbial composition. Pairwise comparisons of beta diversity between groups were further performed using the Kruskal–Wallis test to identify differences in microbial community structure.

Linear discriminant analysis effect size (LEfSe) analysis was performed with the R package “*lefser*” to identify potential discriminatory taxa among the three groups. This approach identifies taxa with statistically significant differences in abundance across groups and estimates their contribution to group discrimination using linear discriminant analysis (LDA), with an LDA score threshold of 4.0.

To assess the specific association of anger with the gut microbiome of diabetic patients, differential microbiome analysis was further focused on the IDM and NIDM groups. The “*MaAsLin2*” R package was used for differential abundance analysis at the genus level. This tool employs generalized linear models to evaluate associations between microbiome features and phenotypes, adjusting for potential confounders, including age, sex, BMI, diabetes duration, and metformin use. Associations with false discovery rate (FDR)-adjusted *p* < 0.05 were considered statistically more robust. The discriminatory performance of the differential microbiome features identified by MaAsLin2 was assessed using receiver operating characteristic (ROC) curves. The discriminatory performance of the differential features identified by MaAsLin2 was further evaluated using an internal validation framework based on ridge logistic regression with leave-one-out cross-validation (LOOCV) and L2 regularization to reduce overfitting.

### 2.4 Metabolomics analysis

Fasting venous blood samples were collected from all participants, including the HC group. Blood metabolites were analyzed using UPLC-MS/MS (Yuan et al., 2012; Want et al., 2010). Samples were refrigerated during transport to the laboratory and then stored at −80 °C until analysis. A 100 μL blood sample was mixed with 400 μL of 80% methanol solution (LC-MS Grade, Thermo Fisher, USA), vortexed, and incubated in an ice bath for 5 min, followed by centrifugation at 15,000*g* at 4 °C for 20 min. The supernatant was diluted to 53% methanol with mass spectrometry-grade water (Merck, Germany), centrifuged again, and the supernatant was collected. Quality control (QC) and blank samples were prepared by mixing equal volumes. Detection was performed using the Vanquish UHPLC system with a Hypersil Gold C18 column (100 × 2.1 mm, 1.9 μm, Thermo Fisher, USA) coupled to a Q Exactive^TM^ HF mass spectrometer (Thermo Fisher, Germany). Raw data files (.raw) were imported into compound discoverer (CD) 3.1 software for processing, including peak alignment, peak extraction, and molecular formula prediction, followed by comparison with mzCloud, mzVault, and Masslist databases.

A total of 26,607 features were initially detected. To obtain a reliable metabolite list, features were first filtered to retain only those matching a confirmed metabolite name. Duplicate identifications were then resolved by prioritizing mzCloud > mzVault > Masslist, and for identical database results, the higher score was retained. For missing values, a multi-step hierarchical imputation strategy within CD 3.1 was used, including matching ions, low-threshold peak detection, Gaussian fitting, or the detection limit. Raw quantification data were normalized by dividing each metabolite intensity by the total metabolite intensity of the corresponding sample and then multiplying by the mean total intensity of the QC samples. Metabolites with a coefficient of variation >30% in QC samples were removed. After these filtering and normalization steps, 970 metabolites were retained for further analysis. Partial least squares discriminant analysis (PLS-DA) was performed, and exploratory metabolite markers were selected based on *p* < 0.05, variable importance in projection (VIP) > 1, and |log_2_ fold change (FC)| > 1. Associations were further assessed using multiple linear regression after adjusting for covariates, and discriminatory performance was assessed using ROC curves.

### 2.5 Differential microbiota–metabolite correlation analysis

Spearman correlation analysis was applied to explore associations between differentially abundant genera and exploratory metabolite markers. Spearman correlation coefficients and corresponding *p* values were calculated using the “ *corr.test*” function in the R package *Hmisc*. Associations with FDR-adjusted *p* < 0.05 were considered statistically more robust.

### 2.6 Functional prediction

To investigate the potential functional implications of anger on the gut microbiome in diabetic patients, Phylogenetic Investigation of Communities by Reconstruction of Unobserved States 2 (PICRUSt2) was applied to predict the KEGG orthologous groups (KO) and Clusters of Orthologous Groups (COG) functional categories based on 16S rRNA gene sequencing data. Differential analysis of the predicted KO and COG categories was performed using MaAsLin2, with adjustments for age, sex, BMI, diabetes duration, and metformin use. KO entries and COG functional categories showing nominally significant differences between the two groups were identified. KEGG pathway enrichment analysis was conducted on KO entries showing nominally significant differences using the “*clusterProfiler*” package with Benjamini–Hochberg FDR correction (adj. *p* < 0.05) to reveal key metabolic pathways and biological processes associated with microbiome functional potential. Functional annotation of COG categories showing nominally significant differences was performed to clarify the functional differentiation of microbiome-encoded proteins.

All statistical analyses were conducted using a two-tailed test, with *p* < 0.05 considered statistically significant.

## 3 Results

### 3.1 Cross-sectional cohort characteristics

Table 1 and Supplementary Table S1 summarize the cohort characteristics for the IDM, NIDM, and HC groups, respectively. The cohort included 29 individuals in the IDM group, 28 in the NIDM group, and 30 in the HC group. No significant differences were observed between the IDM and NIDM groups in body mass index (BMI), Fasting Plasma Glucose (FPG), Hemoglobin A1c (HbA1c), Triglycerides (TG), Total Cholesterol (TC), Uric Acid (UA), High-Density Lipoprotein Cholesterol (HDL-C), Low-Density Lipoprotein Cholesterol (LDL-C), Apolipoprotein A1 (ApoA1), or Apolipoprotein B (ApoB). All variables satisfied the normality assumption based on visual inspection of Q-Q plots. Table 1 also reported the use of antidiabetic medications in the IDM and NIDM groups. Meanwhile, Supplementary Table S1 summarized the cohort differences between the HC group and the 57 patients with T2DM. Except for significant differences in sex, FPG and HbA1c, no other significant differences were observed. Overall, the two diabetic groups were comparable in basic demographic characteristics and key metabolic indicators, providing a balanced basis for subsequent analysis of microbiota and metabolic differences.

**Table 1:** Clinical characteristics of IDM and NIDM participants.

Characteristic	IDM (*N* = 29)	NIDM (*N* = 28)	*p*-value
Age, mean ± SD	55 ± 10	56 ± 10	0.707^a^
Sex, *n* (%)			
Female	15 (51.7%)	10 (35.7%)	0.223^b^
Male	14 (48.3%)	18 (64.3%)	
BMI, mean ± SD	25.6 ± 5.1	25.4 ± 4.4	0.863^a^
Diabetes duration, mean ± SD	6.8 ± 6.6	6.3 ± 4.8	0.771^a^
FPG, mmol/L, mean ± SD	7.67 ± 2.07	7.77 ± 2.12	0.861^a^
HbA1c, %, mean ± SD	7.57 ± 1.33	7.91 ± 1.90	0.437^a^
TG, mmol/L, mean ± SD	2.08 ± 2.73	2.37 ± 2.27	0.668^a^
TC, mmol/L, mean ± SD	4.82 ± 0.92	4.58 ± 1.44	0.451^a^
UA, umol/L, mean ± SD	308 ± 76	294 ± 85	0.518^a^
HDL-C, mmol/L, mean ± SD	1.38 ± 0.42	1.22 ± 0.36	0.141^a^
LDL-C, mmol/L, mean ± SD	2.68 ± 0.77	2.74 ± 1.27	0.814^a^
ApoA1, g/L, mean ± SD	1.51 ± 0.36	1.35 ± 0.26	0.053^a^
ApoB, g/L, mean ± SD	0.96 ± 0.20	0.99 ± 0.35	0.761^a^
Antihyperglycemic agents, *n*			
Ins	8 (22.2%)	7 (17.9%)	
MET	15 (41.7%)	17 (43.6%)	
SGLT2i	1 (2.8%)	4 (10.3%)	
SUs	4 (11.1%)	4 (10.3%)	
DPP-4i	2 (5.6%)	2 (5.1%)	
TZDs	1 (2.8%)	2 (5.1%)	
AGIs	4 (11.1%)	2 (5.1%)	
Glinides	1 (2.8%)	1 (2.6%)	

^a^Welch two sample t-test; ^b^Pearson's Chi-squared test.

IDM, irritable DM; NIDM, non-irritable DM; FPG, fasting plasma glucose; HbA1c, hemoglobin A1c; TG, triglycerides; TC, total cholesterol; UA, uric acid; HDL-C, high-density lipoprotein cholesterol; LDL-C, low-density lipoprotein cholesterol; ApoA1, apolipoprotein A1; ApoB, apolipoprotein B; Ins, insulin; MET, metformin; SGLT2i, sodium-glucose cotransporter-2 inhibitors; SUs, sulfonylureas; DPP-4i, dipeptidyl peptidase-4 inhibitors; TZDs, thiazolidinediones; AGIs, alpha-glucosidase inhibitors; Glinides, glinides.

### 3.2 Gut microbiota structure and diversity

**Figure 2:**
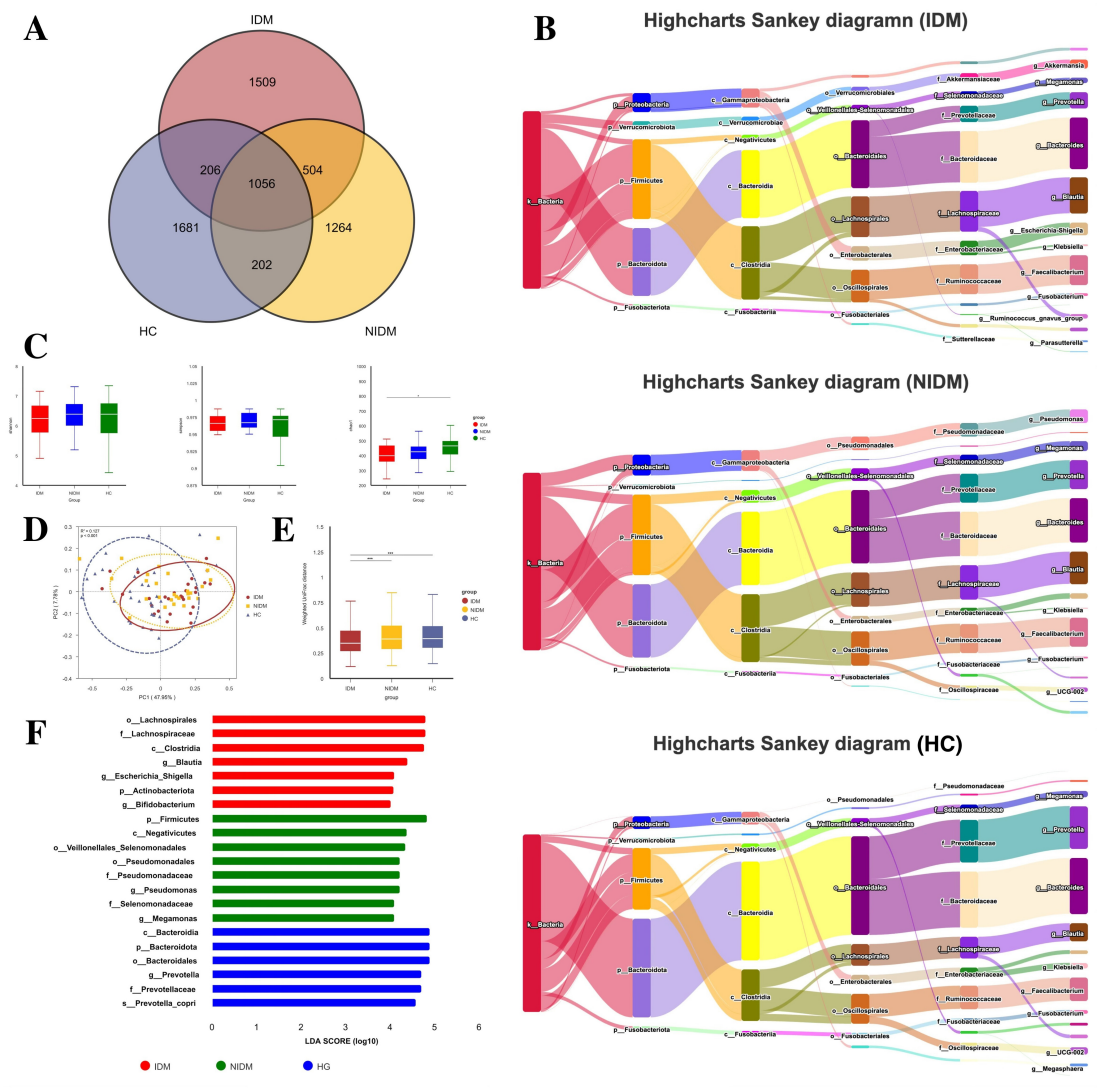
Gut microbiota profile characterization among IDM, NIDM, and HC groups. **(A)** Venn diagram showing the shared and unique amplicon sequence variants (ASVs) of gut microbiota among IDM, NIDM, and HC groups; **(B)** Sankey diagrams showing the relative abundance flow of gut microbiota across different taxonomic levels in the IDM, NIDM, and HC groups. The width of each ribbon is proportional to the relative abundance, illustrating the distribution and proportional relationships of microbial taxa across hierarchical levels; **(C)** Alpha diversity assessment using Shannon, Simpson, and Chao1 indexes; **(D)** principal coordinates analysis (PCoA) plot based on the weighted UniFrac distance matrix, showing the clustering pattern of microbial community structures; **(E)** beta diversity inter-group difference plot presenting the Beta diversity variations among the three groups; **(F)** LEfSe analysis plot showing the potential discriminatory taxa among the three groups. *p < 0.05, **p < 0.01, ***p < 0.001.

A total of 189 genera and 154 species were retained for further analysis. To characterize the group-specific and shared features of the gut microbiome composition across groups, Venn and Sankey diagrams were generated. Figure 2A illustrated the distribution of shared and unique species at the amplicon sequence variant (ASV) level across the groups. ASVs represent distinct sequence variants identified through high-resolution sequencing and capture fine-scale microbial variation within samples. The results indicated differences in the gut microbiome composition among the groups. The IDM, NIDM, and HC groups contained 1,509, 1,264, and 1,681 unique ASVs, respectively. The Sankey diagram (Figure 2B) combined taxonomic hierarchy with flow characteristics to effectively display differences and relationships in the relative abundance across different taxa. At the phylum level, *Firmicutes* and *Bacteroidota* were predominant in all three groups. At the genus level, *Bacteroides, Blautia*, and *Faecalibacterium* were more abundant in the IDM and NIDM groups. The group differences in species composition suggested that anger may be linked to shifts in gut microbial community structure, providing a basis for subsequent diversity quantification and differential abundance analysis.

To further quantify and compare microbial diversity within each group, Chao1, Shannon, and Simpson indices were calculated and compared (Figure 2C, Supplementary Table S2). The Kruskal–Wallis test with *post-hoc* Dunn's test revealed significant differences in species richness (Chao1) only between the IDM and HC groups (*p* = 0.04). No significant differences were observed between NIDM and HC, or between IDM and NIDM groups. For the Shannon and Simpson indices, no statistical significance was found between any pair of groups. These findings suggest that anger may affect the species richness of the gut microbiota in T2DM patients, rather than its evenness or overall diversity.

Building on this, Beta diversity analysis was conducted to examine the differences in microbial community structure between groups. The PCoA plot revealed distinct microbial community patterns among the groups. The PERMANOVA test showed significant differences between the groups (*R*^2^ = 0.127, *p* < 0.001; Figure 2D). Further comparison using the Kruskal-Wallis test showed significant differences between IDM and NIDM (*p* < 0.001), and between IDM and HC (*p* < 0.001; Figure 2E, Supplementary Table S3). Beta diversity analysis indicated that self-reported anger was associated with differences in the overall structure of the gut microbiota in T2DM patients, and this difference was not attributable to diabetes alone. This finding provides support for identifying anger-associated microbial features.

LEfSe was applied to identify potential discriminatory taxa between the IDM, NIDM, and HC groups. Figure 2F showed the 21 discriminatory taxa selected using an LDA score threshold of ≥4, with seven taxa for IDM, eight for NIDM, and six for HC. Specifically, in the IDM group, significantly enriched taxa across different levels included *Lachnospirales, Lachnospiraceae, Clostridia, Blautia, Escherichia Shigella, Actinobacteriota, Bifidobacterium*.

### 3.3 Identification of differentially abundant genera and exploratory metabolite markers

**Figure 3:**
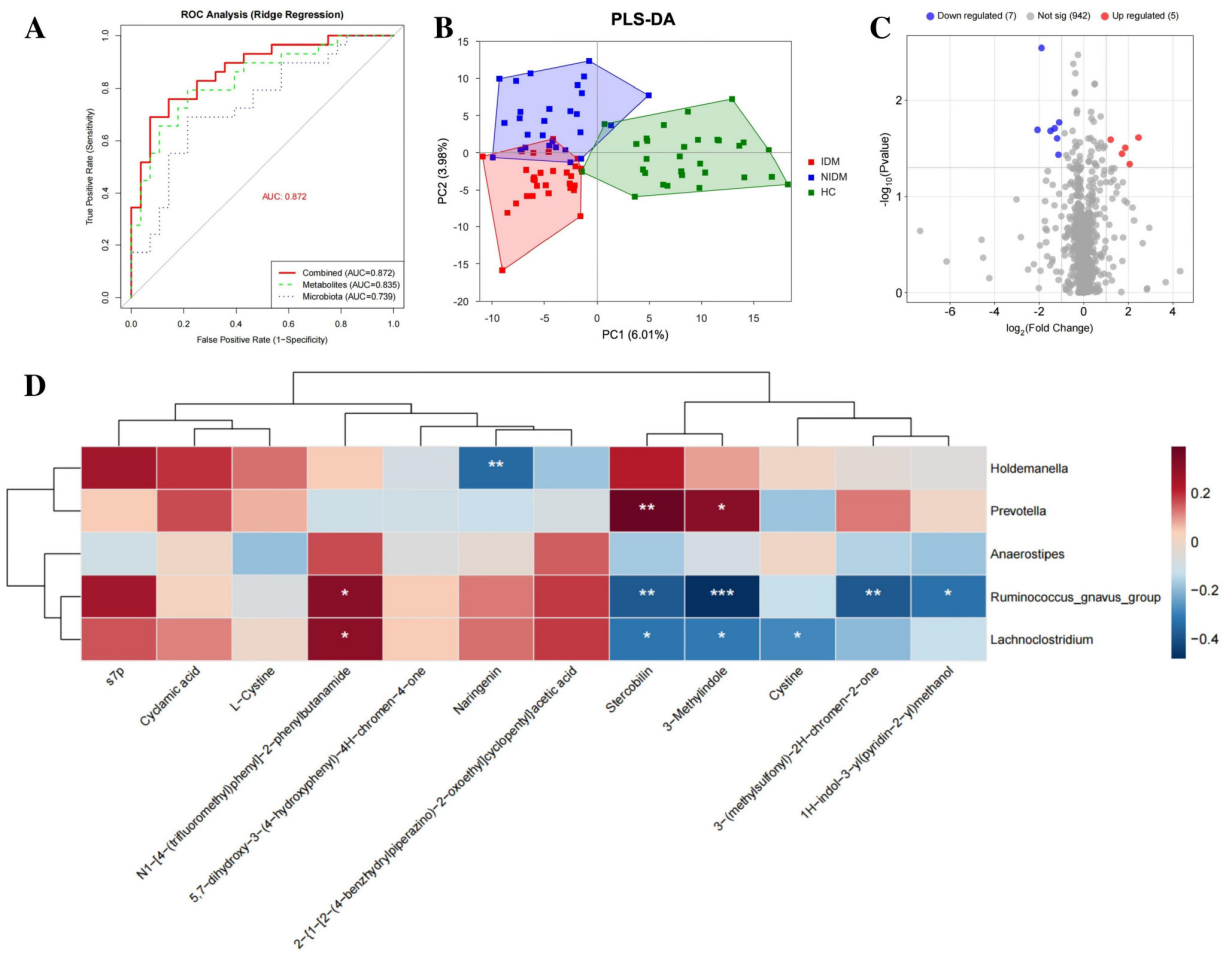
Blood metabolite distribution, differential characteristics, microbiota-metabolite associations, and diagnostic efficacy analysis. **(A)** Ridge Regression-based ROC analysis plot evaluating the diagnostic discriminative efficacy of differentially abundant genera, exploratory metabolite markers, and their combined model between IDM and NIDM groups; **(B)** PLS-DA plot showing the distribution pattern of metabolites in IDM, NIDM, and HC groups; **(C)** Volcano plot of differential metabolites presenting the distribution characteristics between IDM and NIDM groups; **(D)** Spearman correlation heatmap displaying the correlation degree between differentially abundant genera and exploratory metabolite markers. *p < 0.05, **p < 0.01, ***p < 0.001.

To further explore the specific association of anger with the gut microbiome of diabetic patients, we focused on identifying differentially abundant genera between the IDM and NIDM groups. Based on MaAsLin2, after adjusting for confounding factors that may affect microbiome characteristics, we identified five differentially abundant genera: *Holdemanella* (down-regulated in IDM), *Ruminococcus gnavus group* (up-regulated in IDM), *Lachnoclostridium* (up-regulated in IDM), *Anaerostipes* (up-regulated in IDM), and *Prevotella* (down-regulated in IDM). After FDR correction, only *Holdemanella* remained significant (adj. *p* < 0.05), whereas the other four genera showed nominal significance (*p* < 0.05; Supplementary Table S4). The area under the ROC curve (AUC) of the discriminant model based on these five genera was 0.739 (Figure 3A), indicating acceptable discriminatory performance.

The gut microbiome affects the host's physiological and pathological processes primarily through its metabolic products. To systematically reveal related host metabolic changes, untargeted metabolomic profiling was performed on blood samples from the IDM (*n* = 29), NIDM (*n* = 28), and HC (*n* = 30) groups. After strict quality control, all QC samples exhibited Pearson correlation coefficients above 0.98, and *R*^2^ values close to 1, indicating stable performance of the chromatographic-mass spectrometric system (Supplementary Figure S1). PLS-DA revealed clustering differences in metabolic profiles between the groups (Figure 3B). A total of 955 metabolites were identified across 87 samples. Based on predefined criteria (*p* < 0.05, VIP > 1, |log_2_ FC| > 1), 12 metabolites showed nominally significant differences between the IDM and NIDM groups. Compared to the NIDM group, the IDM group showed increased levels of 5 metabolites (5,7-dihydroxy-3-(4-hydroxyphenyl)−4H-chromen-4-one, 2-{1-[2-(4-benzhydrylpiperazino)-2-oxoethyl]cyclopentyl} acetic acid, N1-[4-(trifluoromethyl) phenyl]-2-phenylbutanamide, Naringenin, and 1H-indol-3-yl (pyridin-2-yl)methanol) and decreased levels of seven metabolites [cystine, cyclamic acid, 3-methylindole, stercobilin, S7P, 3-(methylsulfonyl)-2H-chromen-2-one, and L-Cystine; Figure 3C]. The 12 exploratory metabolite markers were mainly classified into flavonoids, indoles, sulfur-containing amino acids, stercobilins, and phosphosugars. After adjusting for potential confounders, multivariate linear regression analysis revealed significant independent associations for eight metabolites and marginally significant associations for four metabolites with the group (Supplementary Table S5). ROC curve analysis indicated that the 12 metabolite markers could effectively classify IDM and NIDM groups, with an AUC of 0.835. Further construction of a combined model incorporating microbial and metabolomic markers revealed an increase in AUC to 0.872, significantly outperforming the models using microbiota or metabolites alone (Figure 3A). The observed microbial and metabolomic differences, together with their combined discriminatory performance, provide exploratory evidence for further investigation of microbiota-metabolite associations and anger-related metabolic regulatory mechanisms.

### 3.4 Correlation analysis of differentially abundant genera and exploratory metabolite markers

**Figure 4:**
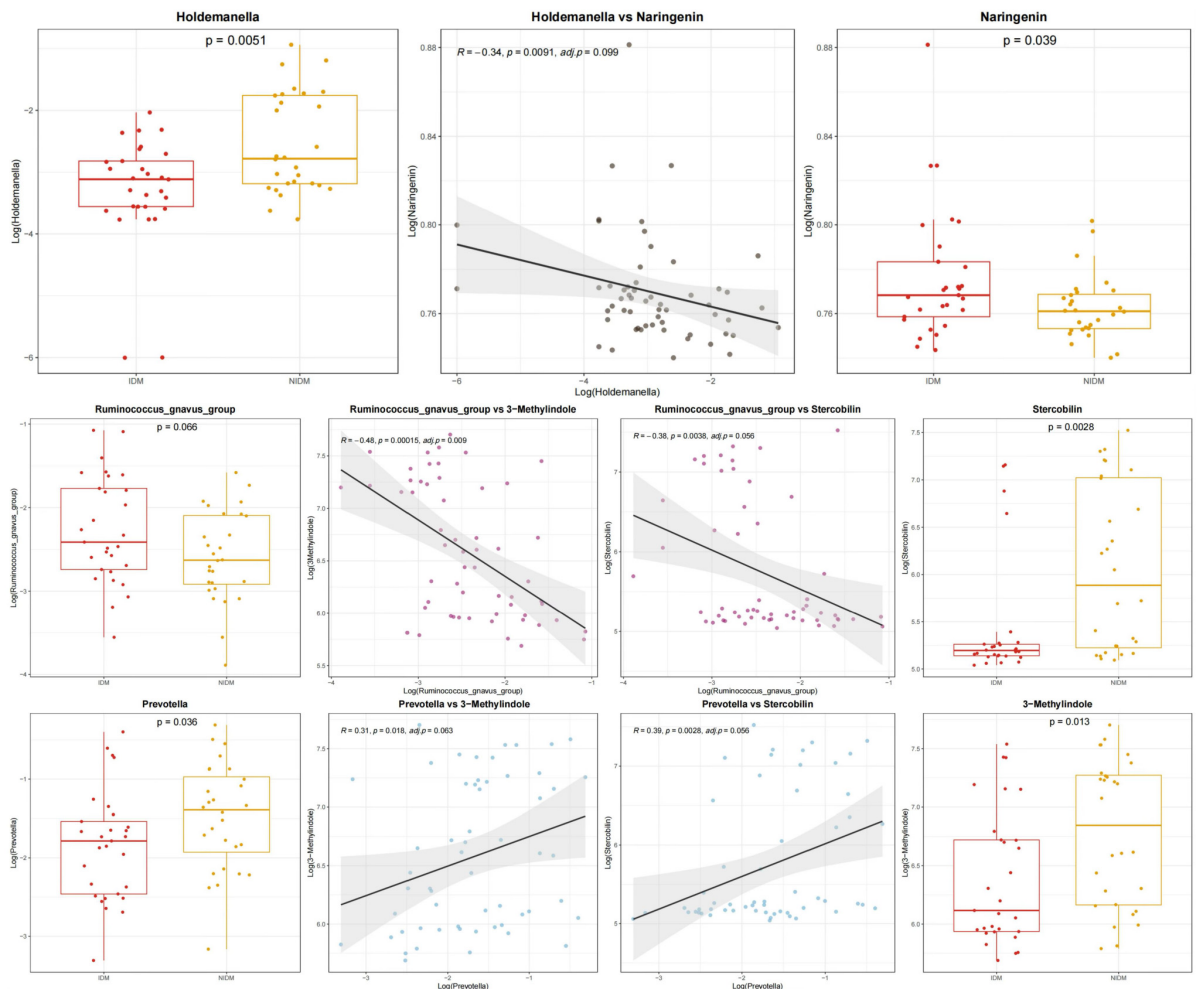
Associations between differentially abundant genera and exploratory metabolite markers in IDM and NIDM groups. Boxplots showing the abundance differences of *Holdemanella, Ruminococcus gnavus* group, *Prevotella*, naringenin, stercobilin, and 3-methylindole between the two groups; scatter plots presenting the Spearman correlation relationships between *Holdemanella, Ruminococcus gnavus* group, *Prevotella* and naringenin, stercobilin, 3-methylindole respectively. adj. *p* indicates *p*-value after FDR multiple testing correction.

To further explore the potential relationships between the five differentially abundant genera and 12 exploratory metabolite markers, Spearman correlation analysis was performed. The analysis revealed 12 statistically significant microbiota-metabolite pairs (Figure 3D), with eight pairs showing negative correlations and four showing positive correlations. Boxplots and scatter plots (Figure 4) further illustrated the inter-group abundance/content differences and correlations of key microbiota-metabolite pairs. The correlation between *Holdemanella* and Naringenin was significant (*r* = −0.48, *p* = 0.009). After FDR correction, the adj. *p* was 0.099, which could be considered a marginal association under an exploratory threshold. Additionally, compared to the NIDM group, the relative abundance of *Ruminococcus gnavus group* showed a marginal increase in the IDM group (*p* = 0.066), while the relative abundance of *Prevotella* significantly decreased (*p* = 0.036). At the metabolite level, the content of stercobilin (*p* = 0.028) and 3-methylindole (*p* = 0.013) were significantly lower in the IDM group. Correlation analysis showed significant positive correlations between the relative abundance of *Prevotella* and both 3-methylindole (r = 0.31, *p* = 0.018) and stercobilin (*r* = 0.39, *p* = 0.003). Conversely, the relative abundance of *Ruminococcus gnavus group* was significantly negatively correlated with both 3-methylindole (*r* = −0.48, *p* < 0.001) and stercobilin (*r* = −0.38, *p* = 0.004; Supplementary Tables S6, S7). After FDR correction, the association between *Ruminococcus gnavus group* and 3-methylindole remained significant (*adj.p* < 0.05), while the associations of *Ruminococcus gnavus group* with stercobilin (*adj.p* = 0.056), *Ruminococcus gnavus group* with 3-(methylsulfonyl)-2H-chromen-2-one (*adj.p* = 0.056), and Prevotella with stercobilin (*adj.p* = 0.056) were considered marginally significant (Supplementary Table S8).

### 3.5 Functional prediction

**Figure 5:**
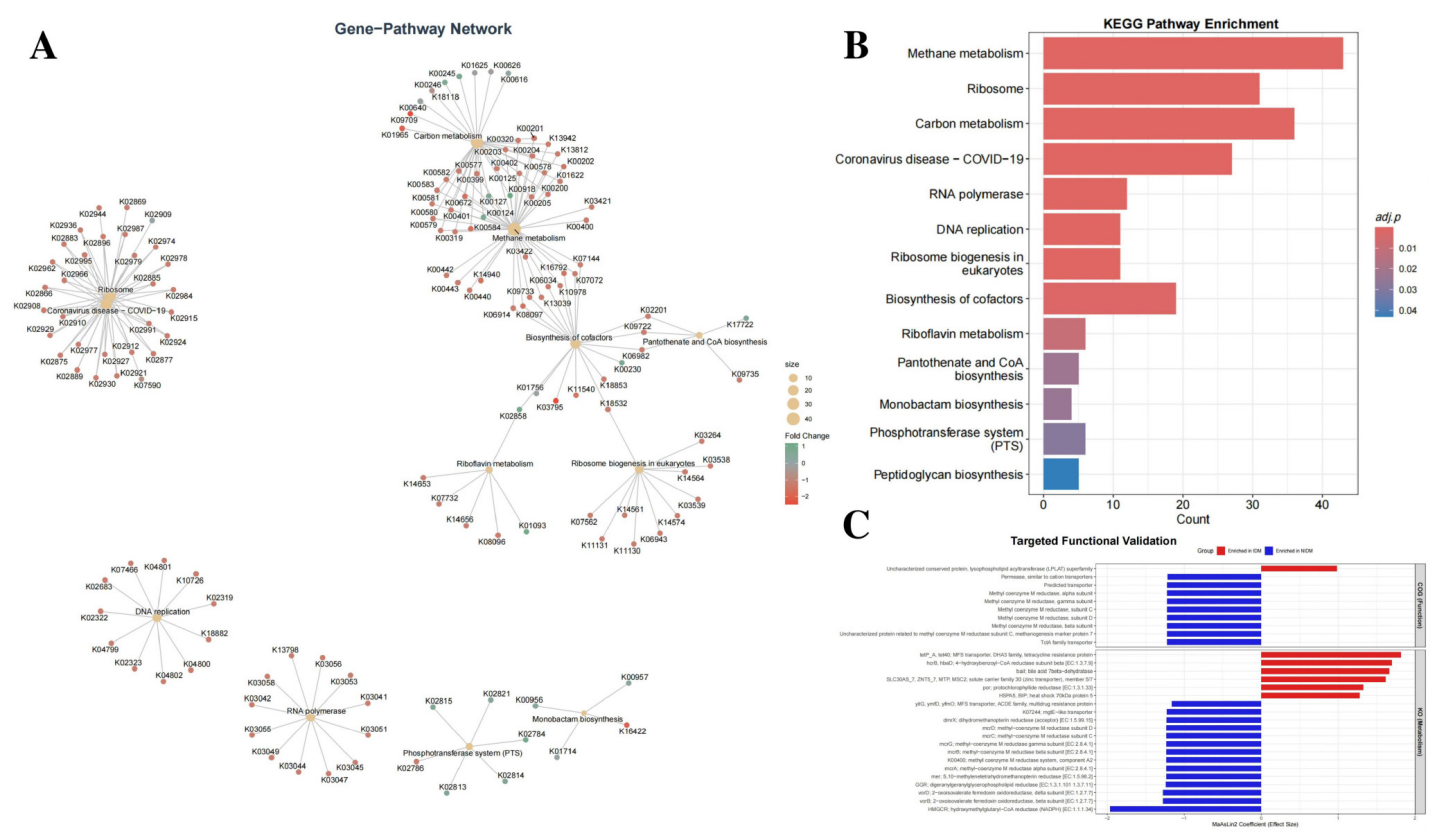
Differential associations and validation analysis of gut microbiota functional pathways. **(A)** KEGG gene-pathway network diagram showing the association relationship between differential KO genes and corresponding KEGG pathways; **(B)** KEGG pathway gene coefficient plot displaying the distribution characteristics of gene coefficients corresponding to different KEGG pathways; **(C)** differential functional validation plot presenting the abundance differences of differential KEGG and COG functions between IDM and NIDM groups based on MaAsLin2 analysis.

To explore the potential functional pathways through which the gut microbiome may be linked to host metabolic status, functional prediction and comparative analyses were performed between the IDM and NIDM groups. First, PICRUSt2 was used to predict the genomic functional potential of the gut microbiota based on 16S rRNA gene sequencing data. This analysis annotated 7312 KOs. To control for potential confounders and identify functional features showing nominally significant differences between the groups, MaAsLin2 analysis was performed. A total of 480 KO entries showing nominally significant differences between the groups were identified. To elucidate the biological processes associated with these differentially represented functional features, KEGG pathway enrichment analysis was conducted for the 480 significant KOs. The top 20 enriched pathways, including methane metabolism, carbon metabolism, pantothenate and CoA biosynthesis, and phosphotransferase system (PTS), are shown in Figures 5A,B.

Additionally, using the same analysis pipeline (PICRUSt2 prediction combined with MaAsLin2 differential analysis), we identified and compared the Clusters of Orthologous Groups of proteins (COG) functional categories to further validate predicted microbiome functional differences from a protein function perspective. Figure 5C showed the top-ranked KO and COG entries showing nominally significant differences identified by MaAsLin2, highlighting the predicted microbial functional features most closely associated with anger in diabetes. Several differential functional entries between the groups were closely linked to key bile acid metabolism steps and related regulatory pathways. Functional prediction analysis revealed significant predicted functional differences in metabolic pathways between the IDM and NIDM groups, consistent with the previously identified differential metabolites and the regulatory pathways of key microbiota.

## 4 Discussion

This study focused on the often-overlooked dimension of self-reported anger-related irritability in T2DM, integrating 16S rRNA gut microbiota sequencing and untargeted metabolomics analyses. The gut microbiota and metabolic phenotypes were compared among the IDM, NIDM, and HC groups, yielding the following key findings. First, significant separation in gut microbiota composition was observed among the three groups, as shown by beta diversity analysis and PCoA visualization, indicating that self-reported anger was associated with overall shifts in gut microbiome composition. Second, focusing on the two diabetes groups, five differentially abundant genera were identified, with a discriminatory AUC of 0.739. After FDR correction, only *Holdemanella* remained significant (*adj.p* < 0.05). Additionally, 12 exploratory metabolite markers were identified with an AUC of 0.835. Spearman correlation analysis revealed 12 nominally significant microbiota-metabolite associations (*p* < 0.05), and after FDR correction, the association between *Ruminococcus gnavus group* and 3-methylindole remained significant (*adj.p* < 0.05). *Holdemanella* also showed a negative correlation with naringenin. Finally, functional prediction analysis showed that the microbiota functional differences between the IDM and NIDM groups were mainly enriched in pathways related to riboflavin metabolism, pantothenate and CoA biosynthesis, monobactam biosynthesis, the phosphotransferase system (PTS) and peptidoglycan biosynthesis. Together, these preliminary and exploratory findings outline key features of the gut microbiota-metabolite interaction network associated with self-reported anger, suggesting potential pathways through which the gut-brain-metabolism axis may be involved in emotion-related metabolic dysfunction in diabetes.

The five differentially abundant genera identified in this study may represent exploratory microbial markers linking emotional disturbances with metabolic diseases. *Holdemanella* has been implicated in both metabolic regulation and emotional modulation. (Romaní-Pérez et al. 2021) demonstrated that *Holdemanella* biformis improved glucose tolerance and restored GLP-1 sensitivity in obese mice, suggesting a beneficial role in metabolic homeostasis. In contrast, (Jiang et al. 2025) reported that in a stress-associated irritable bowel syndrome model, *Holdemanella* biformis was associated with anxiety-like behaviors, indicating that its alteration under stress conditions may contribute to emotional disturbances. These findings suggest that *Holdemanella* may serve as a dual-function microbe linking metabolic and emotional pathways, with its effects potentially dependent on host context. *Lachnoclostridium* has been shown to correlate with the severity of depressive symptoms and was reported to have a potential causal relationship with T2DM risk in a Mendelian randomization study (Sun et al., 2023; Radjabzadeh et al., 2022). *Anaerostipes*, a short-chain fatty acid (SCFA)-producing bacterium, is significantly reduced in abundance in T2DM patients (Balvers et al., 2021). As an SCFA producer, Anaerostipes may be involved in emotional and glucose dysregulation when its abundance is reduced. The *Ruminococcus gnavus group* was one of the differentially abundant taxa showing nominal significance identified in the irritable DM group and may be linked to anger-related metabolic dysfunction in diabetes. Previous studies have reported associations between this bacterium and mood and behavioral abnormalities. The abundance of the *Ruminococcus gnavus group* has been shown to increase significantly in patients with generalized anxiety disorder and is associated with rule-breaking behavior in children with Attention Deficit Hyperactivity Disorder (ADHD) (Lee et al., 2022; Jiang et al., 2018). Additionally, its abundance is significantly higher in T2DM and prediabetes patients compared to healthy individuals, and is consistently associated with the risk of developing T2DM, particularly in those with severe insulin resistance and hyperlipidemia (Xu et al., 2023). As a tryptophan-metabolizing bacterium, *Ruminococcus gnavus* can degrade tryptophan to produce neuroactive metabolites (Williams et al., 2014). Previous studies suggest that it may influence gut-blood-brain barrier permeability (Hoyles et al., 2018), potentially facilitating metabolite transport to the CNS and thus contributing to the regulation of emotional and metabolic processes (Canfora et al., 2019). Furthermore, *Ruminococcus gnavus* has been associated with increased levels of inflammatory factors such as NLRP3 and IL-6, which may promote chronic low-grade inflammation, thereby providing a potential link between emotional stress and diabetes progression (Hong et al., 2024).

For *Prevotella*, previous studies have reported a significant reduction in its abundance in individuals experiencing high emotional stress, accompanied by higher levels of depression, anxiety, and perceived stress (Dong et al., 2024). *Prevotella* is a major SCFA producer that contributes to intestinal barrier maintenance and neurotransmitter modulation. Its abundance has been reported to correlate negatively with the severity of anxiety symptoms and positively with symptom alleviation (Chen et al., 2019). In addition to its association with emotional fluctuations, *Prevotella* abundance is typically reduced in diabetes patients and negatively correlates with HbA1c (Zhao et al., 2024). Additionally, *Prevotella* P2 strains are associated with increased risks of obesity and diabetes (Bah et al., 2025). Potential mechanisms linking Prevotella to emotional and metabolic phenotypes may involve SCFAs, which enhance intestinal barrier integrity, suppress low-grade inflammation, and may act as HDAC inhibitors with possible effects on central neurotransmitter synthesis (Davie, 2003; Yano et al., 2015). This pathway may contribute to the link between anger and metabolic dysfunction in diabetes.

In this study, 12 exploratory metabolite markers were identified, many of which are closely associated with mood regulation and the pathological progression of diabetes, and may act as key signaling molecules in the gut-brain-metabolism axis. For example, animal studies have shown that naringenin can regulate the PPARγ/NLRP3 inflammation pathway, thereby improving both hyperglycemia and depressive-like behavior in diabetic rats, suggesting a potentially important role in the mood-metabolism interaction (El-Marasy et al., 2024). Naringenin has also been shown to exert neuroprotective, anxiolytic, and antidepressant effects by modulating monoaminergic systems, reducing oxidative stress and neuroinflammation, and upregulating BDNF expression (Haidary et al., 2026; Olugbemide et al., 2021). 3-Methylindole, an indole derivative from tryptophan metabolism, has been reported to inhibit neuroinflammation via AhR activation and may influence hepatic glucose output and insulin sensitivity (Rothhammer et al., 2016). This mechanism may involve AhR-mediated cross-talk between central and peripheral organs. Lower 3-methylindole levels in the IDM group may weaken these protective effects, potentially exacerbating the interplay between emotional disturbances and insulin resistance.

Stercobilin has been shown to induce macrophage production of pro-inflammatory cytokines (e.g., TNF-α, IL-1β). Chronic low-grade inflammation is considered a potential mechanism linking emotional stress, HPA axis activation, and impaired blood glucose control (Sanada et al., 2020). Clinical observations have shown that patients with depression and metabolic abnormalities have lower stercobilin levels, which are significantly associated with inflammation and blood glucose markers (Su et al., 2014). Bile acid metabolites such as stercobilin may protect gut barrier integrity by inhibiting NF-κB signaling and modulating tight junction proteins, thereby attenuating inflammation-associated metabolic and emotional disturbances (Huang et al., 2023; Peng et al., 2009). Stercobilin may also influence hypothalamic glucose metabolism through the FXR-FGF19 axis, potentially affecting glucose homeostasis (Liu et al., 2018). Taken together, these microbial and metabolomic features may represent potential targets linking emotional regulation with the pathological progression of T2DM. Their putative multi-pathway regulatory roles provide insight into gut-ecological processes that may underlie the comorbidity between emotional disturbances and metabolic diseases.

Spearman correlation analysis revealed co-variation patterns between *Ruminococcus gnavus group* and *Prevotella* and certain metabolites, supporting the existence of a microbiota-metabolite co-variation network that warrants further causal investigation. Chronic irritability might activate the HPA axis and increase cortisol, which in turn could be associated with enrichment of *Ruminococcus gnavus group* and reduction of *Prevotella* (Gao et al., 2018; Yu et al., 2017). In the tryptophan cycle, *Ruminococcus gnavus* may convert tryptophan to tryptamine through tryptophan decarboxylase, while competing for the substrate, thereby inhibiting the metabolism of indole-producing bacteria (Williams et al., 2014). The depletion of *Prevotella* might further reduce indole precursor synthesis, leading to the downregulation of 3-methylindole. Reduced *Prevotella* abundance might impair secondary bile acid formation, while *Ruminococcus gnavus* could alter bile acid profiles, potentially disrupting FXR/TGR5 feedback and lowering stercobilin (Monteiro-Cardoso et al., 2021; Chen et al., 2018). Hypothetically, reduced 3-methylindole may weaken AhR-mediated anti-inflammatory effects, whereas altered stercobilin may affect insulin signaling via FXR/TGR5 pathways. However, the proposed synergistic inhibition of IRS-1 phosphorylation remains to be tested (Canfora et al., 2019; Rothhammer et al., 2016). The gut-brain-metabolism regulatory network, particularly pathways involving bile acid and tryptophan metabolism, may therefore represent an important direction for future studies on the impact of emotions on metabolic dysregulation. Notably, *Holdemanella* was down-regulated while naringenin was up-regulated in the IDM group. This inverse relationship might reflect a compensatory response to anger-induced stress, possibly aimed at counteracting metabolic or inflammatory challenges. However, the causal direction and underlying mechanisms require further investigation through targeted *in vitro* and *in vivo* studies.

Given the relatively limited sample size of this study and the modest associations between anger and gut microbiota or metabolic phenotypes in patients with T2DM, the magnitude of between-group differences was relatively small. In this study, we primarily performed statistical analyses using raw *p*-values. For the identification of differentially abundant genera, Spearman correlation analysis, and KEGG enrichment analysis, we further indicated results with an adjusted *p*-value below 0.05 alongside raw *p*-values. For the screening of exploratory metabolite markers, we additionally applied FC and VIP thresholds. This analytical strategy is consistent with common practice in hypothesis-generating multi-omics research. It retains nominally significant findings as exploratory differences and associations to provide clues for future investigations, while clearly highlighting those that remain significant after multiple testing correction. This study focuses on the mechanism by which anger may influence metabolic dysregulation in patients with T2DM, a research direction that has rarely been reported and is therefore exploratory in nature. Its aim is to preliminarily reveal potential links through which anger may mediate gut microbiota-related metabolic disturbances in diabetes. The nominally significant findings may provide preliminary and hypothesis-generating directions for future research in this field.

This study has several other limitations. First, anger was assessed using a single-item visual analog scale (VAS). This approach was selected as a pragmatic and rapid exploratory stratification tool, rather than as a validated diagnostic or comprehensive psychometric instrument. While VAS-based measures offer efficiency for brief affective screening in large-scale or multi-omics studies, their limitations must be explicitly recognized. A single-item VAS cannot fully distinguish multidimensional components of anger, including state anger, trait anger, and anger expression or control. It also cannot reliably differentiate anger from related but distinct constructs, such as hostility, depressive irritability, anxiety-related agitation, and broader emotional distress. Consequently, we acknowledge that the observed associations may, in part, reflect these overlapping dimensions of negative emotionality rather than anger specifically. This limitation highlights the need for future studies to incorporate more established psychometric instruments, such as the State-Trait Anger Expression Inventory (STAXI), the PROMIS Anger scales, or other validated multidimensional tools, to enable more rigorous emotional phenotyping in translational multi-omics research. Second, the cross-sectional design of the study can only reveal associations between microbiota, metabolites, and anger or diabetes, without establishing causal relationships. The sample size of this study is relatively small, which may lead to limited statistical power, increased risk of overfitting, and reduced generalizability in the context of multi-omics integration. Furthermore, the relatively small sample size may affect the statistical power, particularly for associations with low-abundance microbiota and metabolites, and these results should be interpreted with caution. A significant sex difference was observed between the HC group and the diabetic groups, which might have contributed to some of the observed differences, although the core analyses focused on the comparison between IDM and NIDM and all multivariable models were adjusted for sex. Additionally, KEGG and COG functional predictions were based on PICRUSt2 inference from 16S rRNA data, which provides indirect estimates of functional potential rather than direct measurement of gene content or expression. Therefore, shotgun metagenomics, metatranscriptomics, or targeted functional assays will be needed to validate these inferred pathways. Finally, although key confounding factors were adjusted for, the study did not fully consider the impact of dietary habits, exercise patterns, and medication duration on gut microbiota and metabolic phenotypes, potentially overlooking important regulatory factors. Given the exploratory nature of this study and the modest statistical robustness of some findings, these results should be interpreted cautiously and require validation in independent cohorts.

## 5 Conclusion

This exploratory pilot investigation focuses on the often-overlooked dimension of anger in T2DM. Through multi-omics analysis, we preliminarily found that differentially abundant genera, such as *Ruminococcus gnavus group* and *Prevotella*, together with exploratory metabolite markers, such as 3-methylindole and stercobilin, were associated with anger-related metabolic dysregulation in T2DM, suggesting potential candidates for future hypothesis-driven investigation. These findings provide a new perspective for understanding the pathophysiology of negative emotions, often not classified as psychiatric disorders, and their comorbidity with metabolic diseases. Additionally, they offer theoretical insights for developing emotion-regulating interventions targeting microbiota or metabolites in diabetes, with potential relevance for both fundamental research and clinical translation. Given the exploratory design, limited sample size, and modest number of FDR-significant findings, the current results should primarily be interpreted as hypothesis-generating observations requiring validation in larger, independently replicated cohorts with more rigorous emotional phenotyping.

## Data Availability

The raw data generated in this study can be found in the NCBI (https://www.ncbi.nlm.nih.gov/), accession PRJNA1479731.
