# Supplementary material for: Integrated gut microbiota and metabolome analyses link anger to metabolic dysregulation in patients with type 2 diabetes mellitus
Source: Front Microbiol. 2026 Jul 9;17:1797695. doi: 10.3389/fmicb.2026.1797695 (PMC13393217; doi:10.3389/fmicb.2026.1797695)
Supplement: Supplementary file 1 [file Supplementary_File_1.docx]

**Online Supplementary Material**

**Title:** Integrated gut microbiota and metabolome analyses link anger to metabolic dysregulation in patients with type 2 diabetes mellitus

**Author:** Xue-li Bao^a,b#^, Ye-xin Chen^a,b#^, Tu-nan Ding^b^, Dan-dan Zhao^b^, Mo-han Sun^b^, Qian-wen Yang^a,b^, Dong-sen Hu^b^, Fang-fang Mo^b^, Gui-xiao Yang^c^, Si-hua Gao^b*^, Jin-kun Ma^d*^, Tian Tian^b*^

*a. Dongzhimen Hospital, Beijing University of Chinese Medicine, Beijing, China;*

*b. Beijing University of Chinese Medicine, Beijing, China;*

*c. Graduate School of China Academy of Chinese Medical Sciences, Beijing, China;*

*d. China Press of Traditional Chinese Medicine Co., Ltd., Beijing, China.*

**# Xue-li Bao, Ye-xin Chen contributed equally to this manuscript.**

**Table S1:** Baseline Characteristics of DM vs. HC participants.

**Table S2:** Inter-group differences of Shannon, Simpson, and Chao1 indices.

**Table S3:** Inter-group differences of beta diversity.

**Table S4:** Results of suggestive differential microbiota screened by MaAsLin2.

**Table S5:** Multivariate Regression Analysis of Differential Metabolites.

**Table S6:** Spearman Correlation Coefficients.

**Table S7:** *p*-Values from Spearman Correlation Analysis.

**Table S8:** *adj.p*-Values from Spearman Correlation Analysis.

**Figure. S1:** Pearson Correlation Heatmap of Quality Control (QC) Samples Demonstrating High Reproducibility of Metabolomic Detection

**Table S1:** Baseline Characteristics of DM vs. HC participants.

| **Characteristic** | **DM (N = 57)** | **HC (N = 30)** | ***p*-value** |
| --- | --- | --- | --- |
| **Age, Mean ± SD** | 55 ± 10 | 54 ± 8 | 0.482^1^ |
| **Sex, n (%)** |  |  | 0.020^2^ |
| **Female** | 25 (43.9%) | 21 (70.0%) |  |
| **Male** | 32 (56.1%) | 9 (30.0%) |  |
| **BMI, Mean ± SD** | 25.5 ± 4.8 | NA ± NA |  |
| **FBG, mmol/L, Mean ± SD** | 7.72 ± 2.08 | 5.19 ± 0.34 | <0.001^1^ |
| **HbA1c, %, Mean ± SD** | 7.74 ± 1.63 | 5.67 ± 0.32 | <0.001^1^ |
| **TG, mmol/L, Mean ± SD** | 2.22 ± 2.50 | 1.74 ± 2.81 | 0.431^1^ |
| **TC, mmol/L, Mean ± SD** | 4.70 ± 1.20 | 4.88 ± 0.70 | 0.375^1^ |
| **UA, umol/L, Mean ± SD** | 301 ± 80 | 263 ± 72 | 0.030^1^ |
| **HDL-C, mmol/L, Mean ± SD** | 1.30 ± 0.40 | 1.45 ± 0.37 | 0.097^1^ |
| **LDL-C, mmol/L, Mean ± SD** | 2.71 ± 1.04 | 2.80 ± 0.60 | 0.589^1^ |
| **ApoA1, g/L, Mean ± SD** | 1.43 ± 0.32 | 1.46 ± 0.29 | 0.650^1^ |
| **ApoB, g/L, Mean ± SD** | 0.97 ± 0.28 | 0.96 ± 0.17 | 0.769^1^ |
| ^1^Welch Two Sample t-test; ^2^Pearson's Chi-squared test | | | |

DM, Diabetes Mellitus; HC, Health control; FPG, fasting plasma glucose; HbA1c, hemoglobin A1c; TG, triglycerides; TC, total cholesterol; UA, uric acid; HDL-C, high-density lipoprotein cholesterol; LDL-C, low-density lipoprotein cholesterol; ApoA1, apolipoprotein A1; ApoB, apolipoprotein B; Ins, insulin; MET, metformin; SGLT2i, sodium-glucose cotransporter-2 inhibitors; SUs, sulfonylureas; DPP-4i, dipeptidyl peptidase-4 inhibitors; TZDs, thiazolidinediones; AGIs, alpha-glucosidase inhibitors; Glinides, glinides.

**Table S2:** Inter-group differences of Shannon, Simpson, and Chao1 indices.

**Inter-group differences of Shannon index**

|  | Difference | pvalue | LCL | UCL |
| --- | --- | --- | --- | --- |
| IDM vs NIDM | -4.63 | 0.494 | -18.06 | 8.79 |
| IDM vs HC | -2.12 | 0.750 | -15.32 | 11.07 |
| NIDM vs HC | 2.51 | 0.709 | -10.81 | 15.83 |

IDM, irritable DM; NIDM, non-irritable DM; HC, Health control.

**Inter-group differences of Simpson index**

|  | Difference | pvalue | LCL | UCL |
| --- | --- | --- | --- | --- |
| IDM vs NIDM | -4.78 | 0.481 | -18.20 | 8.64 |
| IDM vs HC | -2.14 | 0.748 | -15.33 | 11.05 |
| NIDM vs HC | 2.63 | 0.695 | -10.68 | 15.94 |

IDM, irritable DM; NIDM, non-irritable DM; HC, Health control.

**Inter-group differences of Chao1 index**

|  | Difference | pvalue | LCL | UCL |
| --- | --- | --- | --- | --- |
| IDM vs NIDM | -5.02 | 0.448 | -18.13 | 8.08 |
| IDM vs HC | -13.81 | 0.036 | -26.69 | -0.93 |
| NIDM vs HC | -8.79 | 0.182 | -21.79 | 4.21 |

IDM, irritable DM; NIDM, non-irritable DM; HC, Health control.

**Table S3:** Inter-group differences of beta diversity.

|  | Difference | pvalue | LCL | UCL |
| --- | --- | --- | --- | --- |
| IDM vs NIDM | -83.04 | 9e-04 | -132.10 | -33.98 |
| IDM vs HC | -92.56 | 1e-04 | -139.92 | -45.19 |
| NIDM vs HC | -9.52 | 0.699 | -57.78 | 38.75 |

IDM, irritable DM; NIDM, non-irritable DM; HC, Health control.

**Table S4:** Results of suggestive differential microbiota screened by MaAsLin2.

| **Feature** | **coef** | **stderr** | ***p*** | ***adjusted p*** |
| --- | --- | --- | --- | --- |
| *Holdemanella* | -2.29 | 0.60 | <0.001 | 0.049 |
| *Prevotella* | -1.21 | 0.60 | 0.049 | 0.48 |
| *Ruminococcus_gnavus_group* | 1.18 | 0.55 | 0.036 | 0.48 |
| *Anaerostipes* | 0.658046249 | 0.3242542 | 0.048 | 0.48 |
| *Lachnoclostridium* | 0.510243463 | 0.2506453 | 0.047 | 0.48 |

**Table S5:** Multivariate Regression Analysis of Differential Metabolites.

| **Dependent variable** | **Beta^1^** | **95% CI^2^** | ***p*-value** |
| --- | --- | --- | --- |
| Cyclamic acid | -0.34 | -0.68, 0.00 | 0.055 |
| Stercobilin | -0.74 | -1.11, -0.37 | <0.001 |
| L-Cystine | -0.19 | -0.34, -0.03 | 0.023 |
| s7p | -0.21 | -0.42, 0.01 | 0.063 |
| 3-(methylsulfonyl)-2H-chromen-2-one | -0.23 | -0.39, -0.07 | 0.008 |
| Cystine | -0.22 | -0.44, 0.00 | 0.053 |
| 3-Methylindole | -0.43 | -0.75, -0.11 | 0.011 |
| 5,7-dihydroxy-3-(4-hydroxyphenyl)-4H-chromen-4-one | 0.2 | 0.03, 0.37 | 0.028 |
| 1H-indol-3-yl(pyridin-2-yl)methanol | 0.19 | 0.02, 0.36 | 0.033 |
| Naringenin | 0.26 | 0.09, 0.43 | 0.004 |
| N1-[4-(trifluoromethyl)phenyl]-2-phenylbutanamide | 0.39 | 0.00, 0.78 | 0.054 |
| 2-{1-[2-(4-benzhydrylpiperazino)-2-oxoethyl]cyclopentyl}acetic acid | 0.31 | 0.09, 0.53 | 0.009 |

1. Beta, 95%CI, and *p*-value refer to the change in Log10-transformed metabolite levels in the IDM group compared with the NIDM group, with different metabolites as the dependent variable, the IDM vs NIDM grouping as the independent variable, and covariates adjusted for.
2. CI = Confidence Interval

Adjusted for Age, Gender, BMI, Diabetes duration, and Metformin use.

**Table S6:** Spearman Correlation Coefficients.

|  | *Holdemanella* | *Ruminococcus_gnavus_group* | *Lachnoclostridium* | *Anaerostipes* | *Prevotella* |
| --- | --- | --- | --- | --- | --- |
| Cyclamic acid | 0.19 | 0.02 | 0.11 | 0.00 | 0.17 |
| Stercobilin | 0.22 | -0.38 | -0.32 | -0.17 | 0.39 |
| L-Cystine | 0.13 | -0.07 | -0.01 | -0.19 | 0.08 |
| s7p | 0.26 | 0.26 | 0.16 | -0.10 | 0.04 |
| 3-(methylsulfonyl)-2H-chromen-2-one | -0.04 | -0.38 | -0.20 | -0.15 | 0.13 |
| Cystine | 0.01 | -0.13 | -0.28 | 0.00 | -0.18 |
| 3-Methylindole | 0.09 | -0.48 | -0.31 | -0.10 | 0.31 |
| 5,7-dihydroxy-3-(4-hydroxyphenyl)-4H-chromen-4-one | -0.09 | 0.04 | 0.05 | -0.07 | -0.10 |
| 1H-indol-3-yl(pyridin-2-yl)methanol | -0.06 | -0.33 | -0.13 | -0.18 | 0.00 |
| Naringenin | -0.34 | 0.13 | 0.13 | -0.03 | -0.12 |
| N1-[4-(trifluoromethyl)phenyl]-2-phenylbutanamide | 0.04 | 0.32 | 0.31 | 0.17 | -0.11 |
| 2-{1-[2-(4-benzhydrylpiperazino)-2-oxoethyl]cyclopentyl}acetic acid | -0.18 | 0.18 | 0.19 | 0.15 | -0.08 |

**Table S7:** *p*-Values from Spearman Correlation Analysis.

|  | *Holdemanella* | *Ruminococcus_gnavus_group* | *Lachnoclostridium* | *Anaerostipes* | *Prevotella* |
| --- | --- | --- | --- | --- | --- |
| Cyclamic acid | 0.155 | 0.863 | 0.397 | 0.982 | 0.211 |
| Stercobilin | 0.104 | 0.004 | 0.014 | 0.193 | 0.003 |
| L-Cystine | 0.333 | 0.596 | 0.956 | 0.150 | 0.574 |
| s7p | 0.052 | 0.051 | 0.236 | 0.440 | 0.755 |
| 3-(methylsulfonyl)-2H-chromen-2-one | 0.740 | 0.004 | 0.135 | 0.255 | 0.352 |
| Cystine | 0.963 | 0.349 | 0.034 | 0.991 | 0.189 |
| 3-Methylindole | 0.487 | 0.000 | 0.018 | 0.471 | 0.018 |
| 5,7-dihydroxy-3-(4-hydroxyphenyl)-4H-chromen-4-one | 0.506 | 0.748 | 0.711 | 0.604 | 0.468 |
| 1H-indol-3-yl(pyridin-2-yl)methanol | 0.651 | 0.012 | 0.345 | 0.168 | 0.997 |
| Naringenin | 0.009 | 0.349 | 0.323 | 0.807 | 0.372 |
| N1-[4-(trifluoromethyl)phenyl]-2-phenylbutanamide | 0.756 | 0.016 | 0.018 | 0.218 | 0.424 |
| 2-{1-[2-(4-benzhydrylpiperazino)-2-oxoethyl]cyclopentyl}acetic acid | 0.172 | 0.174 | 0.159 | 0.269 | 0.531 |

**Table S8:** *adj.p*-Values from Spearman Correlation Analysis.

|  | *Holdemanella* | *Ruminococcus_gnavus_group* | *Lachnoclostridium* | *Anaerostipes* | *Prevotella* |
| --- | --- | --- | --- | --- | --- |
| Cyclamic acid | 0.476 | 0.942 | 0.644 | 0.997 | 0.503 |
| Stercobilin | 0.416 | 0.056 | 0.099 | 0.483 | 0.056 |
| L-Cystine | 0.603 | 0.770 | 0.997 | 0.476 | 0.765 |
| s7p | 0.223 | 0.223 | 0.524 | 0.677 | 0.856 |
| 3-(methylsulfonyl)-2H-chromen-2-one | 0.856 | 0.056 | 0.476 | 0.545 | 0.603 |
| Cystine | 0.997 | 0.603 | 0.169 | 0.997 | 0.483 |
| 3-Methylindole | 0.696 | 0.009 | 0.099 | 0.689 | 0.099 |
| 5,7-dihydroxy-3-(4-hydroxyphenyl)-4H-chromen-4-one | 0.706 | 0.856 | 0.856 | 0.770 | 0.689 |
| 1H-indol-3-yl(pyridin-2-yl)methanol | 0.814 | 0.099 | 0.603 | 0.476 | 0.997 |
| Naringenin | 0.099 | 0.603 | 0.603 | 0.897 | 0.620 |
| N1-[4-(trifluoromethyl)phenyl]-2-phenylbutanamide | 0.856 | 0.099 | 0.099 | 0.503 | 0.670 |
| 2-{1-[2-(4-benzhydrylpiperazino)-2-oxoethyl]cyclopentyl}acetic acid | 0.476 | 0.476 | 0.476 | 0.556 | 0.724 |

| R^2 | QC1 | QC2 | QC3 | QC4 | QC5 | QC6 | QC7 | QC8 | QC9 | QC10 | QC11 | QC12 | QC13 | QC14 | QC15 | QC16 | QC17 | QC18 | QC19 | QC20 | QC21 | QC22 | QC23 | QC24 | QC25 | QC26 | QC27 | QC28 | QC29 | QC30 |
| --- | --- | --- | --- | --- | --- | --- | --- | --- | --- | --- | --- | --- | --- | --- | --- | --- | --- | --- | --- | --- | --- | --- | --- | --- | --- | --- | --- | --- | --- | --- |
| QC1 | 1 | 0.983 | 0.991 | 0.986 | 0.986 | 0.987 | 0.987 | 0.986 | 0.979 | 0.985 | 0.98 | 0.986 | 0.979 | 0.984 | 0.986 | 0.984 | 0.988 | 0.982 | 0.984 | 0.978 | 0.983 | 0.985 | 0.981 | 0.984 | 0.982 | 0.984 | 0.983 | 0.984 | 0.982 | 0.983 |
| QC2 | 0.983 | 1 | 0.99 | 0.995 | 0.993 | 0.992 | 0.991 | 0.989 | 0.992 | 0.973 | 0.991 | 0.988 | 0.989 | 0.988 | 0.984 | 0.989 | 0.98 | 0.987 | 0.982 | 0.989 | 0.987 | 0.984 | 0.985 | 0.982 | 0.972 | 0.981 | 0.981 | 0.983 | 0.976 | 0.981 |
| QC3 | 0.991 | 0.99 | 1 | 0.993 | 0.993 | 0.994 | 0.993 | 0.993 | 0.99 | 0.986 | 0.99 | 0.992 | 0.988 | 0.99 | 0.991 | 0.991 | 0.99 | 0.99 | 0.99 | 0.988 | 0.99 | 0.99 | 0.988 | 0.989 | 0.984 | 0.988 | 0.988 | 0.989 | 0.985 | 0.988 |
| QC4 | 0.986 | 0.995 | 0.993 | 1 | 0.995 | 0.994 | 0.993 | 0.991 | 0.994 | 0.979 | 0.992 | 0.992 | 0.991 | 0.991 | 0.988 | 0.992 | 0.985 | 0.99 | 0.986 | 0.991 | 0.99 | 0.988 | 0.988 | 0.986 | 0.978 | 0.985 | 0.986 | 0.987 | 0.982 | 0.986 |
| QC5 | 0.986 | 0.993 | 0.993 | 0.995 | 1 | 0.994 | 0.993 | 0.992 | 0.993 | 0.981 | 0.993 | 0.992 | 0.991 | 0.992 | 0.99 | 0.992 | 0.986 | 0.991 | 0.987 | 0.991 | 0.99 | 0.988 | 0.988 | 0.988 | 0.98 | 0.987 | 0.987 | 0.988 | 0.983 | 0.987 |
| QC6 | 0.987 | 0.992 | 0.994 | 0.994 | 0.994 | 1 | 0.994 | 0.993 | 0.994 | 0.984 | 0.993 | 0.993 | 0.992 | 0.991 | 0.991 | 0.993 | 0.988 | 0.992 | 0.989 | 0.992 | 0.991 | 0.99 | 0.99 | 0.989 | 0.982 | 0.988 | 0.989 | 0.99 | 0.985 | 0.989 |
| QC7 | 0.987 | 0.991 | 0.993 | 0.993 | 0.993 | 0.994 | 1 | 0.995 | 0.993 | 0.985 | 0.993 | 0.994 | 0.992 | 0.993 | 0.992 | 0.994 | 0.991 | 0.993 | 0.991 | 0.992 | 0.993 | 0.991 | 0.991 | 0.991 | 0.984 | 0.99 | 0.99 | 0.991 | 0.987 | 0.99 |
| QC8 | 0.986 | 0.989 | 0.993 | 0.991 | 0.992 | 0.993 | 0.995 | 1 | 0.992 | 0.987 | 0.993 | 0.994 | 0.991 | 0.992 | 0.993 | 0.994 | 0.992 | 0.992 | 0.991 | 0.991 | 0.993 | 0.992 | 0.991 | 0.991 | 0.985 | 0.991 | 0.99 | 0.992 | 0.988 | 0.991 |
| QC9 | 0.979 | 0.992 | 0.99 | 0.994 | 0.993 | 0.994 | 0.993 | 0.992 | 1 | 0.979 | 0.995 | 0.993 | 0.994 | 0.992 | 0.99 | 0.994 | 0.985 | 0.993 | 0.988 | 0.994 | 0.991 | 0.989 | 0.99 | 0.987 | 0.978 | 0.987 | 0.987 | 0.989 | 0.982 | 0.987 |
| QC10 | 0.985 | 0.973 | 0.986 | 0.979 | 0.981 | 0.984 | 0.985 | 0.987 | 0.979 | 1 | 0.98 | 0.988 | 0.98 | 0.985 | 0.99 | 0.985 | 0.993 | 0.985 | 0.99 | 0.978 | 0.987 | 0.989 | 0.985 | 0.989 | 0.991 | 0.99 | 0.988 | 0.988 | 0.99 | 0.988 |
| QC11 | 0.98 | 0.991 | 0.99 | 0.992 | 0.993 | 0.993 | 0.993 | 0.993 | 0.995 | 0.98 | 1 | 0.994 | 0.995 | 0.993 | 0.991 | 0.995 | 0.987 | 0.994 | 0.989 | 0.995 | 0.992 | 0.99 | 0.991 | 0.988 | 0.98 | 0.988 | 0.988 | 0.99 | 0.983 | 0.988 |
| QC12 | 0.986 | 0.988 | 0.992 | 0.992 | 0.992 | 0.993 | 0.994 | 0.994 | 0.993 | 0.988 | 0.994 | 1 | 0.993 | 0.994 | 0.995 | 0.995 | 0.993 | 0.994 | 0.993 | 0.993 | 0.994 | 0.994 | 0.993 | 0.993 | 0.987 | 0.993 | 0.992 | 0.993 | 0.989 | 0.992 |
| QC13 | 0.979 | 0.989 | 0.988 | 0.991 | 0.991 | 0.992 | 0.992 | 0.991 | 0.994 | 0.98 | 0.995 | 0.993 | 1 | 0.993 | 0.991 | 0.994 | 0.987 | 0.993 | 0.989 | 0.993 | 0.992 | 0.99 | 0.991 | 0.988 | 0.981 | 0.988 | 0.988 | 0.989 | 0.984 | 0.988 |
| QC14 | 0.984 | 0.988 | 0.99 | 0.991 | 0.992 | 0.991 | 0.993 | 0.992 | 0.992 | 0.985 | 0.993 | 0.994 | 0.993 | 1 | 0.993 | 0.995 | 0.991 | 0.994 | 0.993 | 0.993 | 0.993 | 0.993 | 0.993 | 0.992 | 0.986 | 0.991 | 0.991 | 0.992 | 0.988 | 0.992 |
| QC15 | 0.986 | 0.984 | 0.991 | 0.988 | 0.99 | 0.991 | 0.992 | 0.993 | 0.99 | 0.99 | 0.991 | 0.995 | 0.991 | 0.993 | 1 | 0.994 | 0.994 | 0.993 | 0.994 | 0.991 | 0.994 | 0.994 | 0.993 | 0.994 | 0.991 | 0.994 | 0.994 | 0.994 | 0.992 | 0.994 |
| QC16 | 0.984 | 0.989 | 0.991 | 0.992 | 0.992 | 0.993 | 0.994 | 0.994 | 0.994 | 0.985 | 0.995 | 0.995 | 0.994 | 0.995 | 0.994 | 1 | 0.991 | 0.995 | 0.992 | 0.994 | 0.995 | 0.993 | 0.994 | 0.993 | 0.986 | 0.991 | 0.992 | 0.993 | 0.988 | 0.992 |
| QC17 | 0.988 | 0.98 | 0.99 | 0.985 | 0.986 | 0.988 | 0.991 | 0.992 | 0.985 | 0.993 | 0.987 | 0.993 | 0.987 | 0.991 | 0.994 | 0.991 | 1 | 0.991 | 0.994 | 0.986 | 0.993 | 0.994 | 0.991 | 0.994 | 0.992 | 0.994 | 0.993 | 0.993 | 0.993 | 0.994 |
| QC18 | 0.982 | 0.987 | 0.99 | 0.99 | 0.991 | 0.992 | 0.993 | 0.992 | 0.993 | 0.985 | 0.994 | 0.994 | 0.993 | 0.994 | 0.993 | 0.995 | 0.991 | 1 | 0.993 | 0.994 | 0.994 | 0.994 | 0.994 | 0.992 | 0.987 | 0.992 | 0.992 | 0.993 | 0.988 | 0.993 |
| QC19 | 0.984 | 0.982 | 0.99 | 0.986 | 0.987 | 0.989 | 0.991 | 0.991 | 0.988 | 0.99 | 0.989 | 0.993 | 0.989 | 0.993 | 0.994 | 0.992 | 0.994 | 0.993 | 1 | 0.989 | 0.993 | 0.995 | 0.993 | 0.994 | 0.991 | 0.993 | 0.993 | 0.993 | 0.992 | 0.993 |
| QC20 | 0.978 | 0.989 | 0.988 | 0.991 | 0.991 | 0.992 | 0.992 | 0.991 | 0.994 | 0.978 | 0.995 | 0.993 | 0.993 | 0.993 | 0.991 | 0.994 | 0.986 | 0.994 | 0.989 | 1 | 0.993 | 0.99 | 0.993 | 0.989 | 0.981 | 0.989 | 0.989 | 0.99 | 0.984 | 0.99 |
| QC21 | 0.983 | 0.987 | 0.99 | 0.99 | 0.99 | 0.991 | 0.993 | 0.993 | 0.991 | 0.987 | 0.992 | 0.994 | 0.992 | 0.993 | 0.994 | 0.995 | 0.993 | 0.994 | 0.993 | 0.993 | 1 | 0.994 | 0.994 | 0.994 | 0.988 | 0.993 | 0.993 | 0.994 | 0.991 | 0.994 |
| QC22 | 0.985 | 0.984 | 0.99 | 0.988 | 0.988 | 0.99 | 0.991 | 0.992 | 0.989 | 0.989 | 0.99 | 0.994 | 0.99 | 0.993 | 0.994 | 0.993 | 0.994 | 0.994 | 0.995 | 0.99 | 0.994 | 1 | 0.994 | 0.994 | 0.992 | 0.994 | 0.994 | 0.994 | 0.992 | 0.994 |
| QC23 | 0.981 | 0.985 | 0.988 | 0.988 | 0.988 | 0.99 | 0.991 | 0.991 | 0.99 | 0.985 | 0.991 | 0.993 | 0.991 | 0.993 | 0.993 | 0.994 | 0.991 | 0.994 | 0.993 | 0.993 | 0.994 | 0.994 | 1 | 0.993 | 0.988 | 0.991 | 0.992 | 0.992 | 0.988 | 0.993 |
| QC24 | 0.984 | 0.982 | 0.989 | 0.986 | 0.988 | 0.989 | 0.991 | 0.991 | 0.987 | 0.989 | 0.988 | 0.993 | 0.988 | 0.992 | 0.994 | 0.993 | 0.994 | 0.992 | 0.994 | 0.989 | 0.994 | 0.994 | 0.993 | 1 | 0.992 | 0.994 | 0.995 | 0.994 | 0.993 | 0.994 |
| QC25 | 0.982 | 0.972 | 0.984 | 0.978 | 0.98 | 0.982 | 0.984 | 0.985 | 0.978 | 0.991 | 0.98 | 0.987 | 0.981 | 0.986 | 0.991 | 0.986 | 0.992 | 0.987 | 0.991 | 0.981 | 0.988 | 0.992 | 0.988 | 0.992 | 1 | 0.992 | 0.991 | 0.991 | 0.993 | 0.992 |
| QC26 | 0.984 | 0.981 | 0.988 | 0.985 | 0.987 | 0.988 | 0.99 | 0.991 | 0.987 | 0.99 | 0.988 | 0.993 | 0.988 | 0.991 | 0.994 | 0.991 | 0.994 | 0.992 | 0.993 | 0.989 | 0.993 | 0.994 | 0.991 | 0.994 | 0.992 | 1 | 0.993 | 0.994 | 0.994 | 0.995 |
| QC27 | 0.983 | 0.981 | 0.988 | 0.986 | 0.987 | 0.989 | 0.99 | 0.99 | 0.987 | 0.988 | 0.988 | 0.992 | 0.988 | 0.991 | 0.994 | 0.992 | 0.993 | 0.992 | 0.993 | 0.989 | 0.993 | 0.994 | 0.992 | 0.995 | 0.991 | 0.993 | 1 | 0.995 | 0.993 | 0.995 |
| QC28 | 0.984 | 0.983 | 0.989 | 0.987 | 0.988 | 0.99 | 0.991 | 0.992 | 0.989 | 0.988 | 0.99 | 0.993 | 0.989 | 0.992 | 0.994 | 0.993 | 0.993 | 0.993 | 0.993 | 0.99 | 0.994 | 0.994 | 0.992 | 0.994 | 0.991 | 0.994 | 0.995 | 1 | 0.993 | 0.995 |
| QC29 | 0.982 | 0.976 | 0.985 | 0.982 | 0.983 | 0.985 | 0.987 | 0.988 | 0.982 | 0.99 | 0.983 | 0.989 | 0.984 | 0.988 | 0.992 | 0.988 | 0.993 | 0.988 | 0.992 | 0.984 | 0.991 | 0.992 | 0.988 | 0.993 | 0.993 | 0.994 | 0.993 | 0.993 | 1 | 0.994 |
| QC30 | 0.983 | 0.981 | 0.988 | 0.986 | 0.987 | 0.989 | 0.99 | 0.991 | 0.987 | 0.988 | 0.988 | 0.992 | 0.988 | 0.992 | 0.994 | 0.992 | 0.994 | 0.993 | 0.993 | 0.99 | 0.994 | 0.994 | 0.993 | 0.994 | 0.992 | 0.995 | 0.995 | 0.995 | 0.994 | 1 |

**Figure S1:** Pearson Correlation Heatmap of Quality Control (QC) Samples Demonstrating High Reproducibility of Metabolomic Detection
